# Efforts to Improve Immunization Coverage during Pregnancy among Ob-Gyns

**DOI:** 10.1155/2016/6120701

**Published:** 2016-01-26

**Authors:** Katherine M. Jones, Sarah Carroll, Debra Hawks, Cora-Ann McElwain, Jay Schulkin

**Affiliations:** ^1^Department of Research, American College of Obstetricians and Gynecologists, 409 12th Street SW, Washington, DC 20024, USA; ^2^Department of Psychology, American University, 4400 Massachusetts Avenue NW, Washington, DC 20016, USA; ^3^Practice Division, American College of Obstetricians and Gynecologists, 409 12th Street SW, Washington, DC 20024, USA

## Abstract

*Background.* Influenza and Tdap vaccines are vital factors for improving maternal and neonatal health outcomes.* Methods.* A prospective, longitudinal study was conducted to determine whether the American College of Obstetricians and Gynecologists' (ACOG's) efforts to increase ob-gyn use of their immunization toolkits and vaccination administration were successful. Pre- and postintervention questionnaires were mailed to a random sample of 1,500 ACOG members between August 2012 and July 2015.* Results.* Significantly more postintervention survey ob-gyns reported that they received the immunization toolkits than preintervention survey ob-gyns (84.5% versus 67.0%, *p* < .001). The large majority of ob-gyns from both surveys (76.9% versus 78.9%) reported that they offered or planned to offer influenza vaccinations to their patients for the 2012-2013 and 2014-2015 flu seasons. Postintervention survey respondents were significantly more likely than preintervention survey participants to report that they routinely offer Tdap vaccinations to all patients during pregnancy (76.8% versus 59.3%, *p* < .001).* Conclusion.* ACOG's efforts to improve ob-gyn use of immunization toolkits and vaccine administration appear to have been successful in several ways. ACOG's toolkits are an example of an effective intervention to overcome barriers to offering vaccines and help improve influenza and Tdap immunization coverage for pregnant women.

## 1. Introduction

Vaccinations are essential components of preconception, prenatal, and postpartum care and of improving maternal and neonatal health for a number of infectious diseases. The Centers for Disease Control and Prevention (CDC) Advisory Committee on Immunization Practices (ACIP) and the American College of Obstetricians and Gynecologists (ACOG) currently recommend two immunizations for all pregnant women without contraindication, inactivated influenza and adult-type tetanus toxoid, reduced diphtheria toxoid, and acellular pertussis (Tdap) [1, 2]. Additionally, ACIP and ACOG advise other family members and individuals in close contact with the newborn to receive influenza and Tdap vaccination (“cocooning”) to further protect the infant [2, 3].

Pregnant women and infants are at increased risk of influenza-related morbidity and mortality and adverse pregnancy outcomes [4]. Influenza vaccination during pregnancy, regardless of pregnancy trimester, can significantly help prevent influenza virus infection among pregnant women and their infants younger than six months of age [5, 6]. Despite public health recommendations, national estimates for influenza immunization coverage during pregnancy have been historically low [5, 7]. According to the CDC, only 52.2% of women reported receiving influenza vaccination before or during pregnancy throughout the 2013-2014 influenza season [6].

Pertussis (whopping cough) is an acute, prolonged respiratory illness caused by the organism* Bordetella pertussis*. Rates of pertussis in the United States have been gradually increasing since 1976, with major epidemics of pertussis occurring over the last several years [2]. Waning vaccine-induced immunity is the key contributing factor to the persistence of the disease, particularly in adults and adolescents [8]. However, those at the greatest risk are young infants who have the highest rates of morbidity, complication, hospitalization, and mortality associated with the disease [9, 10]. Studies have shown that parents, older siblings, other family members and relatives living in the household, and individuals who were close contacts were the source of pertussis transmission to young infants in 75%–85% of the cases. Parents, particularly mothers, were the transmission source more than 50% of the time [11]. Immunity against pertussis does not develop until infants have received three doses of diphtheria and tetanus toxoids and acellular pertussis (DTaP) vaccine, usually by six months of age. Thus, infants under the age of six months rely on passively acquired maternal antibodies for protection [12]. In order for the infant to receive adequate levels of pertussis antibodies, ACIP and ACOG recommend Tdap vaccination to all pregnant women during each pregnancy, regardless of prior Tdap vaccination, between 27 and 36 weeks of gestation, and to other family members and individuals in close contact with the newborn [1, 2, 13]. If Tdap vaccination is not given during pregnancy, and the woman has never received Tdap, it should be given postpartum. National estimates indicate that only 9.8% of pregnant women report receiving Tdap vaccination [14].

Physician recommendations for and administration of vaccines have been shown to be the strongest predictors of vaccine receipt among patients [15]. As the primary health care provider for most women during pregnancy, obstetrician-gynecologists (ob-gyns) can and should play a critical role in administering influenza and Tdap vaccinations to women during pregnancy [1, 3, 5]. Previous studies demonstrate that the majority of ob-gyns believe screening for vaccine-preventable diseases and offering immunizations to pregnant women are within their professional responsibilities [16]. Moreover, the majority of ob-gyns know that influenza and Tdap immunizations (89.9% and 58.6%, resp.) are safe to administer during pregnancy, and most ob-gyns (84.5%) agree that pregnant women should receive annual influenza vaccination [16]. Despite ob-gyns' overall positive attitudes toward immunizations, reported rates of influenza and Tdap vaccination coverage vary widely among ob-gyns (66.8%–79.6% and 29.9%, resp.) [17]. Of even greater concern, national estimates indicate that a much smaller percentage of pregnant women report having received influenza and Tdap vaccinations compared to the percentage of ob-gyns who report having offered the vaccines [14], thus warranting further investigation. One study examining ob-gyns' immunization practices found that the majority of ob-gyn respondents (79.6%) believed educational tools for clinicians and patient educational materials should be a priority for ACOG [18].

In an effort to increase the efficacy of these immunization toolkits and ob-gyn use of toolkit materials and immunization administration, ACOG recently revised and revamped its toolkits based on feedback received from four focus groups that met at the 2013 and 2014 ACOG Annual Meeting. The purpose of this pre- and postintervention questionnaire study was to examine whether ACOG's efforts to improve the usefulness of its immunization toolkits were successful.

## 2. Method

### 2.1. Sample and Study Design

A prospective, longitudinal study was conducted to assess the impact of ACOG's efforts to increase ob-gyn use of ACOG's immunization toolkits and vaccination administration. Between August 2012 and March 2013, the ACOG Immunization Department distributed three immunization toolkits to ACOG's general membership. Following the distribution of the third toolkit, a preintervention questionnaire was sent out to a random sample of 1,500 ACOG members. After data collection concluded for the preintervention questionnaire, three revised toolkits (the “intervention”) were sent to ACOG's general membership between September 2013 and September 2014. In October 2014, following the distribution of the third revised toolkit, a postintervention questionnaire was sent to 1,370 participants of the original 1,500 sample.

Revisions to the toolkits included updating the clinical information and revising the wording of some materials based on feedback from focus groups with ACOG members. ACOG also increased the promotion to members through electronic notifications such as e-mail, ACOG newsletters, and ACOG's Immunization for Women website in addition to promotion through partner organizations. These toolkit revisions were expected to provide supplemental information to ob-gyns, who administer influenza and Tdap vaccinations, in order to improve the usefulness of the immunization toolkits.

A questionnaire on ob-gyn practices and opinions related to immunizations was developed by a team of researchers in the Immunization Department at ACOG familiar with this subject. The preintervention questionnaire contained 24 questions regarding physicians' receipt and use of ACOG's immunization toolkits, immunization resources needed, general immunization practice patterns, barriers to offering vaccinations, and physician use of ACOG's Immunization for Women website. Demographic questions included gender, year of birth, and state/territory of primary practice. Some items were added to the postintervention questionnaire to gather more detailed information regarding participants' demographic background, their patient population, and their use of ACOG's most recently distributed immunization toolkits. New demographic questions included the participants' number of years in practice, type of practice, primary medical specialty, practice location, primary race/ethnicity of patient population, and primary type of patient insurance. The revised postintervention questionnaire contained a total of 34 questions. Questions included yes/no, check boxes, forced choice, and Likert-scales. The questionnaire was constructed to be completed in approximately 5–10 minutes.

The ACOG Immunization Department has been distributing toolkits on vaccinations to its members since 2011 and has sent a total of seven toolkits to date. These toolkits contain resources to help educate ob-gyns and their patients on immunizations for influenza, Tdap, and human papillomavirus (HPV) and to provide physicians with tools for integrating immunizations into routine care. Each toolkit is designed based upon existing ACOG guidance primarily derived from ACOG Committee Opinions. Toolkits feature corresponding materials, including a letter from ACOG's Vice President of Practice Activities, encouraging providers to use the resources in the toolkit; frequently asked questions (FAQs) handouts for patients; a Physician Script; coding information relevant to specific vaccines; and partner materials such as Vaccine Information Statements and sample standing orders from the CDC and Immunization Action Coalition (IAC).

### 2.2. Preintervention Study

ACOG membership includes 95% of board-certified obstetrician-gynecologists in the United States. In January 2013, a random sample of 1,500 active ACOG members was selected to participate in this study. Participation was voluntary, with no compensation offered to participants. Participants were sent an electronic flyer alerting them that they would shortly receive an invitation to participate in an electronic questionnaire on ob-gyns' immunization practices. The online survey was conducted using Real Magnet^*®*^, and the purpose, risks, and benefits of the study were outlined in an e-mail containing the live survey link. Up to six reminder e-mails including a link to the electronic questionnaire were sent between February and March 2013 to nonresponders. Participants were not provided with supplementary information regarding the topic of the survey and were instructed not to look up any additional information.

In April 2013, a paper questionnaire was mailed to all nonresponders of the electronic survey (*n* = 1,403). The purpose, risks, and benefits of the study were outlined in an accompanying cover letter. Two subsequent mailings were sent to nonresponders between May and June 2013. Finally, a shortened letter version of the questionnaire, which contained 10 of the original survey items, was sent to a randomly selected sample of nonresponders (*n* = 300) in September 2013. The letter responses were used to assess a potential nonresponse bias and were not included in data analysis. The electronic questionnaire remained open until data collection for the paper mailing ended in October 2013; thus, participants who received the paper questionnaire could elect to complete either the online or paper survey. Participants were instructed to only complete the survey one time, and responses were tracked using deidentified participant ID numbers.

### 2.3. Postintervention Study

In October 2014, 1,370 participants from the original sample were sent an electronic flyer alerting them that they would receive an invitation to participate in the follow-up study (otherwise known as “postintervention study”) on immunization practices among ob-gyns. One hundred thirty participants from the original sample were not included in the follow-up study because they were no longer active members of ACOG. Members who did not participate in the preintervention questionnaire were permitted to participate in the postintervention questionnaire. The online survey was conducted using Real Mail^*®*^, and the purpose, risks, and benefits of the study were outlined in an e-mail containing a live link to the survey. Up to six reminder e-mails including a link to the online survey were sent between October and December 2014 to nonresponders. Participants were not provided with supplementary information regarding the topic of the survey and were instructed not to look up any additional information.

In February 2015, a paper questionnaire was mailed to all nonresponders of the electronic survey (*n* = 1,245). The purpose, risks, and benefits of the study were outlined in an accompanying cover letter. Two subsequent mailings were sent to nonresponders between April and May 2015. Finally, a shortened letter version of the questionnaire, identical to the one used in the preintervention study, was sent to a randomly selected sample of nonresponders (*n* = 300) in June 2015. The letter responses were used to assess for potential nonresponse bias and were not included in data analysis. The electronic questionnaire remained open until data collection for the paper mailings ended in July 2015; thus, participants who received the paper survey could elect to complete either the online or paper questionnaire. Participants were instructed to only complete the survey one time, and responses were tracked using deidentified participant ID numbers. In the follow-up study, all participants were given the option to opt out of completing the survey, and these participants were removed from the total sample size.

### 2.4. Data Analysis

Data were analyzed using a statistical software package (IBM SPSS Statistics^*®*^ 20.0, IBM Corp^*©*^, Armonk, NY). The study was approved by the ACOG Institutional Review Board. Completion of the online survey or return of the completed paper questionnaire indicated informed consent to participate in the study. Descriptive statistics were computed for measures used in the analyses and reported as mean values ± standard deviation. Chi-square tests were performed for categorical and comparative analyses. One-way analysis of variance (ANOVA) was used to compare group means of continuous variables. Nonparametric statistics were computed for comparative analyses of Likert-scale variables. Comparative results were reported as preintervention study % versus postintervention study %. Findings were reported as significant at *p* < .05.

## 3. Results

### 3.1. Preintervention Study

One hundred thirty-one participants completed the electronic survey, 272 participants returned the paper questionnaire, and 31 participants completed the shortened letter version questionnaire, resulting in a total response rate of 29.3%. Nineteen questionnaires were judged invalid (i.e., provider retired or provider was unreachable by mail); these participants were thus excluded from analysis. Responses to the letter questionnaire did not differ significantly from those of the electronic or paper surveys. Letter responses were excluded from data analysis because of the abbreviated questions found in the letter.

ACOG's total membership in 2013 consisted of 30,015 female members (52.5%) and 27,160 male members (47.5%). Among respondents, 203 were female (59.0%) and 141 were male (41.0%). Males were significantly older than females (males, mean age = 55.19 years ± 10.29 years; females, mean age = 45.19 years ± 9.75 years; *F*(1,337) = 81.90, *p* < .001). Respondents were from the District of Columbia, Puerto Rico, and every state in the United States except Delaware, North Dakota, and Wyoming.

### 3.2. Postintervention Study

One hundred and one participants completed the electronic survey, 186 participants returned the paper questionnaire, and 12 participants completed the shortened letter version questionnaire, resulting in a total response rate of 24.0%. Forty-seven questionnaires were judged invalid (i.e., provider retired, provider opted out, or provider was unreachable by mail); these participants were thus excluded from analysis. Responses to the letter questionnaire did not differ significantly from those of the electronic or paper surveys. Letter responses were excluded from data analysis because of the abbreviated questions found in the letter.

Among respondents, 182 were female (64.1%) and 102 were male (35.9%). Males were significantly older than females (males, mean age = 55.51 years ± 9.63 years; females, mean age = 46.18 years ± 9.05 years; *F*(1,279) = 65.77, *p* < .001). Respondents were from the District of Columbia and every state in the United States except Alaska, Delaware, New Hampshire, New Mexico, North Dakota, and Wyoming. Additional demographic information for postintervention study participants can be found in Table 1. The demographic information provided by the postintervention study is similar to the demographic characteristics of ACOG's full membership [19].

### 3.3. Pre- versus Postintervention Study Comparative Analysis

#### 3.3.1. ACOG Immunization Toolkits

Significantly more ob-gyns from the postintervention study (84.5%) reported that they received the immunization toolkits than ob-gyns from the preintervention study (67.0%) (*χ* ^2^ (2, *N* = 681) = 26.77, *p* < .001). Of those who indicated that they had received the toolkits, an average of 87.3% of respondents from both studies reported that they reviewed the toolkit materials. Among postintervention study participants, ob-gyns in group practice (90.3%) were significantly more likely to report that they received the toolkits mailings compared to those in private practice (59.5%) or other types of practice (85.3%) (e.g., community hospital or university faculty and practice) (*χ* ^2^ (4, *N* = 282) = 25.57, *p* < .001).

Providers were asked to indicate the extent to which they planned to use the immunization toolkit resources. Participant responses from both studies are detailed in Table 2. More postintervention study than preintervention study ob-gyns reported already using all of the toolkit resources, except the ACOG Immunization for Women website; however, these results did not reach statistical significance.

Physicians' frequency of toolkit use was assessed. The most frequently used (i.e., “weekly use”) toolkit items reported in pre- and postintervention studies were the Flu FAQ Tear Pad (28.9% versus 31.2%) and the Tdap FAQ Tear Pad (26.7% versus 31.0%). The least frequently used (i.e., “never use”) toolkit resources reported were the Physician Script (58.7% versus 59.2%), Coding Guide (51.3% versus 56.3%), and Immunization for Women website (45.9% versus 52.6%).

#### 3.3.2. Immunization Resources

**Figure 1:**
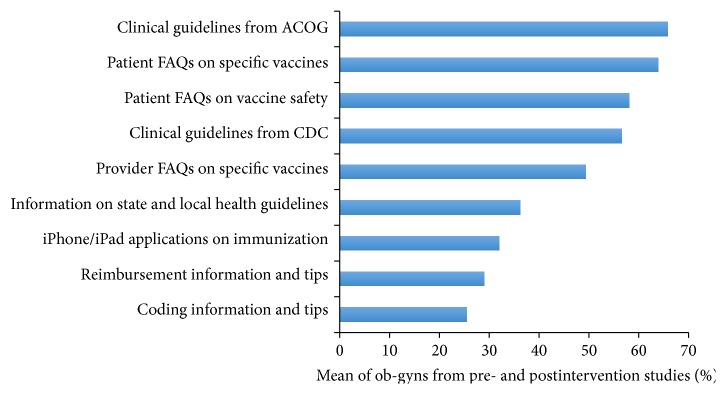
Resources ob-gyns indicated would be most valuable in ACOG's next immunization toolkit.

Providers were asked about their opinions regarding which immunization resources they would find most useful in future immunization toolkits. The immunization resources most frequently selected as valuable in both pre- and postintervention studies were clinical guidelines from ACOG (71.2% versus 58.0%), patient FAQs on specific vaccines (61.3% versus 67.7%), patient FAQs on vaccine safety (54.9% versus 62.6%), and clinical guidelines from the CDC (58.8% versus 53.3%) (Figure 1). Three statistically significant differences were found between these pre- and postintervention study responses (Table 3). The immunization resources reported the least frequently as valuable in the pre- and postintervention studies were not identical; thus, the combined mean responses are reported: videos on immunization (4.4%), webinars on immunization (5.1%), CD-ROMS on vaccinations (5.2%), postgraduate courses (6.6%), and information provided at ACOG Annual District Meetings (6.6%).

#### 3.3.3. Immunization Practices Patterns

Ob-gyns' immunization practice patterns were examined. The use of standing orders for immunizations and the routine administration of Tdap vaccinations during pregnancy appear to be improving. Significantly more providers from the postintervention study (46.6%) than the preintervention study (36.5%) reported that they use standing orders for immunizations in their practices (*χ* ^2^ (1, *N* = 627) = 6.55, *p* = .011). Additionally, postintervention study respondents (76.8%) were significantly more likely than preintervention study participants (59.3%) to report that they routinely offer Tdap vaccinations to all patients during pregnancy (*χ* ^2^ (4, *N* = 612) = 30.55, *p* < .001). Among providers who did not report offering Tdap to all pregnant patients, the majority indicated that they either offer Tdap to their patients postpartum (10.5%) or recommend and refer patients to other local providers (18.3%).

Approximately one-quarter of pre- and postintervention study respondents reported that they have assigned a staff member to be the vaccine coordinator of their practice (23.0% versus 26.1%) or always use a needs assessment with patients to determine what vaccinations they need at the time of their appointment (20.4% versus 27.0%). The large majority of ob-gyns from both studies reported that they offered or planned to offer influenza vaccinations to their patients for the 2012-2013 and 2014-2015 flu seasons (76.9% versus 78.9%). Among physicians who did not offer or plan to offer influenza vaccinations, 97.2% of preintervention study providers and 92.9% of postintervention study providers reported that they would recommend them to their patients or refer patients to local vaccine clinics or providers. Participants were also asked whether they receive annual influenza vaccination. Significantly more respondents from the postintervention study (96.1%) than the preintervention study (90.7%) indicated that they receive an annual flu vaccine (*χ* ^2^ (2, *N* = 678) = 7.44, *p* = .024).

Preintervention study physicians who reported that they annually receive a flu vaccine were significantly more likely to offer Tdap immunizations to all of their pregnant patients (62.5%) (*χ* ^2^ (8, *N* = 342) = 42.09, *p* < .001) compared to physicians who reported that they do not receive annual influenza vaccination. These differences were not present in the postintervention study. However, significant differences based on practice type and primary medical specialty were noted in the postintervention study. Providers in solo practice were less likely to offer Tdap vaccination to all patients during pregnancy (55.9%), administer flu vaccines (53.7%), and use standing orders for immunizations (12.8%) compared with providers in group ob-gyn practice (78.3%, 81.0%, and 46.5%, resp.) or providers practicing in other settings (e.g., university faculty and practice, community hospitals) (84.1%, 87.7%, and 59.5%, resp.) (*p* = .002, *p* < .001, and *p* < .001, resp.). Additionally, physicians who identified their primary medical specialty as general obstetrics and gynecology and obstetrics only were more likely to administer influenza vaccines to their patients (85.6% and 75.0%, resp.) and use standing orders for immunizations (51.5% and 66.7%, resp.) than providers who identified their primary medical specialty as gynecology only (50.0% and 22.7%, resp.) or “other” (i.e., reproductive endocrinology/infertility, maternal/fetal medicine, urogynecology, and gynecologic oncology) (64.4% and 29.5%, resp., *p* < .001).

Lastly, ob-gyns were surveyed about whether they require their staff to receive immunizations for influenza, Hepatitis B, and Tdap. Postintervention study physicians (86.2%) were significantly more likely than preintervention study physicians (78.1%) to report that they required their staff to receive an annual influenza vaccine (*χ* ^2^ (2, *N* = 641) = 6.52, *p* = .011). Responses to the other vaccination questions did not differ significantly between pre- and postintervention study providers. The majority of participants from both studies reported that they require their staff to receive Hepatitis B immunization (62.4% versus 62.1), while slightly less than half of the providers require their staff to receive Tdap vaccination (42.5% versus 49.1%).

#### 3.3.4. Barriers to Offering Immunizations

Ob-gyns were asked to rank their top three barriers to offering immunizations in their offices and the top two most common reasons their patients provide for declining vaccinations. While the top three most frequently reported barriers remained the same for pre- and postintervention studies (inadequate reimbursement, cost, and lack of patient interest), several significant differences were found between the two studies regarding the percentage of respondents who endorsed some of the listed barriers (Table 4). Preintervention study providers were significantly more likely than those of the postintervention study to indicate that cost and lack of access to patient records were barriers to providing immunizations, while postintervention study respondents were more likely to report that time and lack of patient interest were barriers.

Several other demographic differences were observed in the postintervention study. A larger number of ob-gyns who reported practicing in suburban and rural locations indicated that cost (44.7% and 48.4%, *p* < .001) and inadequate reimbursement (52.8% and 51.6%, *p* = .012) were obstacles than clinicians who reported practicing in urban locations (20.7% and 34.2%). Significant demographic differences based upon practice type and primary medical specialty were also found for reported barriers to offering immunization and common reasons patients decline vaccinations. Providers in solo practice were more likely to report cost (48.8%, *p* = .005) and inadequate reimbursement (70.7%, *p* < .001) and least likely to report inadequate time (17.1%, *p* = .038) and concerns about vaccine safety (2.4%, *p* = .013) as barriers than providers in group practice (38.1%, 47.1%, 33.5%, and 22.6%, resp.) or other types of practices (e.g., university faculty and practice, community hospitals) (20.3%, 24.6%, 40.6%, and 18.8%, resp.). Providers who identified their primary medical specialty as obstetrics only (66.7%, *p* = .037) were more likely to report lack of staff as a barrier than providers who identified their primary medical specialty as general obstetrics and gynecology (16.9%), gynecology only (13.6%), or “other” (30.0%) (i.e., reproductive endocrinology/infertility, maternal/fetal medicine, urogynecology, and gynecologic oncology).

According to pre- and postintervention study participants, the top two most common reasons patients provide for declining vaccinations are safety concerns (84.2% versus 78.5%; (*χ* ^2^ (1, *N* = 659) = 3.48, *p* = .062)) and the belief that they do not need vaccines (70.4% versus 80.6%; (*χ* ^2^ (1, *N* = 658) = 8.77, *p* = .003)). While ob-gyns indicated that their patients express concerns over vaccine safety, the majority of respondents from both studies (64.4% versus 76.5%; (*χ* ^2^ (1, *N* = 617) = 10.42, *p* = .001)) estimated that less than one-third of their patients decline vaccinations after their recommendations. Responses from both of these questions suggest that the described concerns are mostly patient concerns; 96.1% of preintervention study providers and 93.9% of postintervention study providers who reported concerns for vaccine safety as a barrier to offering immunizations also selected it as a common reason that patients decline vaccinations.

#### 3.3.5. Immunization for Women Website and Text4baby Program

Ob-gyn awareness and use of the ACOG Immunization for Women website and the Text4baby program were assessed. Text4baby is a free mobile educational service designed for pregnant women to promote maternal and child health through text messaging. No significant differences were found between pre- and postintervention study respondents for any of these variables. Less than one-quarter of pre- (19.0%) and post- (22.1%) intervention study providers reported that they had ever visited ACOG's Immunization for Women website. The majority of pre- and postintervention study ob-gyns reported that they never refer staff (77.3% versus 74.1%), fellow ob-gyns (82.1% versus 80.8%), or patients (76.3% versus 68.4%) to ACOG's Immunization for Women website. Responses to this question did not differ by physician age or gender. Similarly, most ob-gyns were unfamiliar with the Text4baby program (72.7% versus 69.5%). Younger physicians (pre (*χ* ^2^ (4, *N* = 326) = 24.83, *p* < .001); post (*χ* ^2^ (4, *N* = 275) = 22.61, *p* < .001)) and female physicians (pre (*χ* ^2^ (1, *N* = 333) = 6.00, *p* = .014); post (*χ* ^2^ (1, *N* = 276) = 4.15, *p* = .042)) were more likely to be familiar with Text4baby. Among ob-gyns who were familiar with Text4baby, slightly less than half of the pre- and postintervention study respondents (47.1% versus 41.7%) recommended the program to patients who are pregnant or have children.

## 4. Discussion

Findings from this study indicate that ACOG's efforts to improve their immunization resources were successful in many ways. More ob-gyns from the postintervention study reported receiving the immunization toolkits than respondents from the preintervention study. This may be attributed to the more robust promotional campaign that accompanied the second round of immunization toolkits. It is also possible that the increase of postintervention respondents resulted from some type of Hawthorne effect whereby ob-gyns were made aware of the toolkit purely by participating in the preintervention study. Additionally, a greater number of postintervention study providers reported already using all of the immunization toolkit resources (except the Immunization for Women website); however, these results were not statistically significant. The most frequently used toolkit materials reported in both studies were the Flu FAQ Tear Pad and the Tdap FAQ Tear Pad.

The percentage of physicians who reported offering Tdap vaccination to all women during pregnancy increased significantly from 59% to 77% between pre- and postintervention studies. However, these numbers are much higher than those found in the existing literature (30%), indicating that further research is warranted to clarify accurate estimates of Tdap coverage among ob-gyns [18]. The large majority of providers in both studies reported offering influenza vaccines to their patients during the 2013-2014 and 2014-2015 influenza seasons. While our findings align with previously reported rates of influenza administration among ob-gyns [17, 20], an important discrepancy should be noted. According to previously reported national estimates, far fewer pregnant women report receiving influenza and Tdap immunizations than the number of ob-gyns in our study who reported offering them [14, 15]. However, published numbers specifically on Tdap immunization rates are several years old and reflect the rates before ACOG published its recommendations in 2013 to offer the vaccine during every pregnancy [1]. More recent data in states like Wisconsin demonstrate much higher rates of Tdap vaccination during pregnancy than the dreadful “2.6%” that is so commonly referenced [14, 21]. It is possible that ob-gyn respondents are overestimating the proportion of pregnant patients being vaccinated or that ob-gyns respondents of our questionnaires were more likely to routinely administer influenza vaccination or recommend and refer their patients to receive it compared to ob-gyns who did not respond to this study. This discrepancy may also be partially explained by a small percentage of pregnant women who refuse vaccine administration, although research shows that when women are offered vaccination, the majority tends to accept it [15, 22].

Several barriers to offering immunizations were identified by participants. In support of previous findings [16–18], frequently reported obstacles to vaccine administration were financial concerns (cost and inadequate reimbursement), lack of patient interest, lack of time, and inadequate storage for vaccines and supplies. Efficacious interventions are necessary to combat these barriers and improve influenza and Tdap immunization coverage for pregnant women. ACOG recommends several strategies to help ob-gyns prevent missed opportunities for vaccination among pregnant women. These include designating a vaccine coordinator and backup coordinator in their practices to order and receive vaccines, ensure proper storage of vaccines, and be familiar with appropriate billing codes for reimbursement; incorporate needs assessments to determine each pregnant woman's immunization status and administration of indicated vaccinations; and use standing orders to ensure that indicated vaccinations can be administered to all pregnant women without an individual physician order [1]. Discouragingly, less than half of the ob-gyns from the pre- and postintervention studies reported using standing orders for immunizations, and less than one-quarter of participants indicated that they use needs assessments or have assigned a vaccine coordinator within their practices. Other strategies that have been shown to improve immunization rates include educating pregnant women about the maternal and neonatal benefits of immunizations, recommending and offering on-site vaccine administration during pregnancy, and utilizing prompts to help providers and their staff easily identify vaccine-eligible obstetric patients [1, 15, 17, 23]. Improvements in health care policies are essential to help deliver reliable reimbursement to vaccine providers and to curb the high costs of ordering and storing immunizations [24].

One of the limitations to this study is the relatively low response rate. The low response rate may indicate a lack of physician interest in this topic. In order to increase the response rate, multiple mailings and a simplified questionnaire were utilized. It is also possible that characteristics of respondents are different from those of nonrespondents, although nonresponse bias analysis did not reveal statistically significant differences for comparison variables. The simplified questionnaire offered a sufficient amount of content-relevant questions that would assert that those who responded and those who did not respond held similar attitudes towards vaccination during pregnancy. Lastly, these data are based on physician recall and could not be checked through chart review or other methods.

Improving immunization coverage among pregnant women has numerous health benefits for mothers, their infants, and society. While it appears that influenza and Tdap administration rates are increasing among ob-gyns, several barriers to offering immunizations persist. It is crucial to help providers overcome these obstacles in order to ensure that these vaccinations become a routine part of obstetric health care.

## Figures and Tables

**Table 1:** Demographic characteristics of postintervention study respondents.

Characteristics	Percentage (%)
Years since completion of residency	
21–30 years	33.6
11–20 years	28.7
5–10 years	16.1
<5	11.9
Type of practice	
Large group (4+ partners)	43.2
Solo private practice	14.7
University full-time faculty & practice	13.3
Small group (2-3 partners)	10.9
Community hospital full-time	7.7
One partner	3.9
Others	3.9
Community hospital part-time	1.4
Military/government	1.1
Primary medical specialty	
General ob-gyn	74.8
Gynecology only	8.0
Maternal/fetal medicine	7.3
Reproductive endocrinology/infertility	5.6
Gynecologic oncology	2.4
Obstetrics only	1.4
Urogynecology	0.3
Practice location	
Suburban	47.9
Urban, noninner city	25.9
Urban, inner city	15.0
Rural	10.8
Military	0.3
Professional self-identification	
Both primary care physician and specialist	47.9
Specialist	46.9
Primary care physician	5.2
Patient race	
White, non-Hispanic	63.7
Multiracial	16.5
White, Hispanic	10.2
African American, non-Hispanic	3.2
African American, Hispanic	2.1
Asian/Pacific Islander	1.1
American Indian/Alaska native	0.7
Patient insurance	
Private (including HMO, IPO, military)	70.9
Medicaid/Medicare	26.3
Uninsured	2.8

**Table 2:** The extent to which ob-gyns plan to use immunization toolkit resources.

	Already use (%)		Plan to use (%)		Will not likely use (%)		Definitely will not use (%)	
	Pre	Post	Pre	Post	Pre	Post	Pre	Post
Flu FAQ Tear Pad	37.7	44.5	30.0	21.6	21.8	23.3	10.5	10.6
Tdap FAQ Tear Pad	36.1	44.7	31.0	20.8	22.4	23.9	10.6	10.6
Vaccine Safety Tear Pad	30.7	36.5	32.7	21.6	26.0	29.7	10.6	12.2
Immunization for Women website	17.3	15.6	40.3	30.7	31.9	39.9	10.5	13.8
Coding Guide	14.2	16.0	27.6	21.1	40.2	40.8	18.0	22.1
Physician Script	12.4	18.4	21.8	13.2	46.2	46.2	19.7	22.2

FAQ, frequently asked question; Tdap, tetanus-diphtheria-acellular pertussis.

**Table 3:** Statistically significant differences between pre- and postintervention study providers.

Variable	Preintervention study (%)	Postintervention study (%)	*p* value
Received ACOG's immunization toolkit mailings^†^	67.0	84.5	<.001
Valuable immunization resources to include in future toolkit mailings			
Clinical guidelines from ACOG^†^	71.2	58.0	.001
Coding information and tips^†^	30.7	18.0	<.001
Reimbursement information and tips^†^	15.2	9.4	<.001
Barriers to offering immunizations			
Cost^†^	45.5	34.8	.006
Time^*∗*^	25.4	33.0	.036
Lack of access to patient records^*∗*^	7.5	3.7	.048
Lack of patient interest^*∗*^	29.9	37.5	.043
Use standing orders for immunizations^*∗*^	36.5	46.6	.011
Routinely offer Tdap to all pregnant patients^†^	59.3	76.8	<.001
Common reasons patients decline vaccinations			
They do not think they need vaccines^†^	70.4	80.6	.003
Percentage of patients that decline vaccinations			
Less than one-third^†^	64.4	76.5	.001
Receive annual influenza vaccination themselves^*∗*^	90.7	96.1	.024
Require staff to receive annual influenza vaccination^*∗*^	78.1	86.2	.011

ACOG, American College of Obstetricians and Gynecologists; Tdap, tetanus-diphtheria-acellular pertussis.

^*∗*^ *p* < .05, ^†^ *p* < .01.

**Table 4:** Barriers to offering immunizations among ob-gyns.

Barrier	Overall % of ob-gyns who agreed		
	Preintervention study	Postintervention study	*p* value
Inadequate reimbursement	51.4	44.6	.085
Cost^†^	45.5	34.8	.006
Lack of interest from patients^*∗*^	29.9	37.5	.043
Lack of time^*∗*^	25.4	33.0	.036
Lack of storage for vaccine/supplies	24.2	18.0	.059
Concerns about vaccine safety	18.5	18.4	.959
Lack of staff	16.7	19.5	.363
Participating in immunization registries	10.5	9.0	.514
Lack of access to patient records^*∗*^	7.5	3.7	.048

^*∗*^ *p* < .05, ^†^ *p* < .01.
